# Transcranial Brain Stimulation for Neurodevelopmental Disorders: Neuromodulation and Neurodevelopmental Disorders

**DOI:** 10.31661/gmj.vi.3782

**Published:** 2025-08-05

**Authors:** Mohammad Hossein Salemi

**Affiliations:** ^1^ Department of Psychology, University of Tehran, Tehran, Iran

**Keywords:** Transcranial Electrical Stimulation, Transcranial Magnetic Stimulation, Neuromodulation, Neurodevelopmental Disorders

## Abstract

Neurodevelopmental disorders (NDDs) are characterized by cognitive, behavioral, and emotional challenges that significantly impact quality of life. Despite advances in pharmacological and behavioral interventions, many individuals exhibit partial or limited responses, highlighting the need for innovative therapeutic strategies. Non-invasive brain stimulation (NIBS) techniques, particularly transcranial electrical stimulation (TES) and transcranial magnetic stimulation (TMS), have emerged as promising approaches to modulate neural circuits underlying these conditions. Beyond neural modulation, these techniques offer potential clinical benefits, such as improving cognitive and behavioral outcomes in individuals with NDDs, thereby addressing treatment gaps in conventional therapies. While TES primarily alters cortical excitability through electric fields, TMS induces direct neuronal firing via magnetic fields, allowing distinct applications tailored to specific conditions.This review examines the mechanisms, applications, and limitations of TES, such as transcranial direct current stimulation (tDCS), transcranial alternating current stimulation (tACS), and TMS, including repetitive TMS (rTMS) and theta-burst stimulation.

## Introduction

Neurodevelopmental disorders (NDDs) encompass a range of conditions, including autism spectrum disorder (ASD), attention-deficit/hyperactivity disorder (ADHD), Tourette syndrome, and learning disabilities, which emerge early in development and persist throughout life [1]. These disorders often impair critical domains such as cognition, behavior, communication, and motor control, leading to substantial challenges in education, social interactions, and daily functioning [2]. The prevalence of NDDs varies, with studies estimating about 19% of children and adolescents aged 3 to 17 years in the United States [3].

At 8 years old, 23.9% of children with public insurance and 11.0% of those with private insurance had been diagnosed with one or more neurodevelopmental disorders.[4] While traditional interventions focus on managing symptoms, they often fall short in addressing the underlying neural dysfunctions, paving the way for innovative approaches like TES and TMS. [5].

Transcranial electrical stimulation (TES) and transcranial magnetic stimulation (TMS), as key non-invasive brain stimulation (NIBS) techniques, have attracted attention for their ability to modulate neural activity and facilitate neuroplasticity, presenting innovative avenues for addressing neurodevelopmental disorders [6][7]. TES techniques, including transcranial direct current stimulation (tDCS) and transcranial alternating current stimulation (tACS), deliver weak electrical currents to the scalp, influencing cortical excitability in a polarity-specific manner [8]. Similarly, TMS employs magnetic fields to induce electric currents in specific brain regions, enabling targeted stimulation of neural circuits. The non-invasive, adaptable nature of TES and TMS, combined with their ability to target specific neural substrates, makes them particularly promising for treating the underlying dysfunctions in neurodevelopmental disorders [9][10].

The growing body of research exploring the application of TES and TMS in NDDs has demonstrated promising results [7]. Studies have highlighted improvements in social communication deficits in ASD, attention regulation in ADHD, and motor tic suppression in Tourette syndrome following NIBS interventions [11][12][13]. However, the field remains constrained by several challenges, including limited understanding of the underlying mechanisms, variability in study designs, and ethical considerations [14]. This review aims to provide a comprehensive evaluation of the impact of TES and TMS on neurodevelopmental disorders. This article seeks to advance the understanding of NIBS as a promising therapeutic modality for NDDs.

## Mechanisms of TES and TMS

The therapeutic potential of TES and TMS lies in their ability to modulate neural activity and influence neuroplasticity [6][7]. While both techniques share the goal of altering brain function non-invasively, their mechanisms of action differ significantly, making them suitable for different therapeutic targets and clinical scenarios [5][11]. Figure 1 illustrates the primary mechanisms of action for tDCS and TMS in modulating brain activity.

### Mechanisms of TES

TES influences brain function through several key mechanisms. Table-1 demonstrates the main mechanisms of TES. Primarily, it modulates neuronal excitability by altering the resting membrane potential of neurons [15][16] Its ability to induce long-term potentiation (LTP) or long-term depression (LTD) highlights its role in promoting neuroplasticity, which is crucial for learning and adaptive behavioral changes [17]. For instance, anodal stimulation tends to depolarize neurons, increasing their likelihood of firing, while cathodal stimulation hyperpolarizes them, reducing excitability[18][19] . These effects are polarity-dependent and form the basis of techniques like tDCS [16]. Another mechanism involves phase synchronization, where techniques like transcranial tACS apply alternating currents at specific frequencies to entrain oscillatory activity, aligning endogenous brain rhythms with the external current [15]. This frequency-specific modulation is particularly relevant for disorders involving disrupted neural oscillations, such as ADHD and ASD [12][20].

Beyond direct neuronal effects, TES also facilitates stochastic resonance, introducing low-level noise to amplify weak neural signals, improving overall signal detection and processing [21]. This is particularly evident in transcranial random noise stimulation (tRNS)[22]. Subthreshold modulation is another subtle mechanism, where TES influences neural circuits without directly triggering action potentials, effectively "priming" networks for enhanced function [12]. For example, subthreshold modulation primes the motor cortex, enhancing responsiveness to motor training, as seen in rehabilitation studies targeting stroke recovery [21]. Furthermore, TES can modulate neurovascular coupling, indirectly improving blood flow and metabolism in targeted brain regions[21][23]. Lastly, electric fields generated during TES affect ion channels, receptor activity, and neurotransmitter release, impacting both neurons and glial cells. These mechanisms underscore the versatility of TES in enhancing cognition, motor control, and neurorehabilitation [23].

### Mechanisms of TMS

TMS uses electromagnetic induction to create a magnetic field that penetrates the skull, generating electric currents in the brain to directly stimulate neurons [24][25]. Table-2 demonstrates the main mechanisms of TMS. Unlike TES, TMS can induce action potentials, allowing precise, region-specific activation or inhibition of targeted brain regions [26]. Depending on the frequency of stimulation, TMS can either enhance cortical excitability (high-frequency stimulation) or suppress it (low-frequency stimulation) [25]. For instance, high-frequency TMS enhances activity in the dorsolateral prefrontal cortex, which is critical for executive functioning deficits in ADHD, while low-frequency TMS can suppress hyperactivity in motor regions implicated in Tourette syndrome [27][28]. A notable advancement, theta-burst stimulation (TBS), applies pulses in bursts mimicking natural brain rhythms, achieving potent and sustained neuroplastic changes in a shorter duration [29]. TMS is particularly effective in accessing deeper cortical and subcortical structures, enabling the modulation of circuits implicated in neurodevelopmental disorders, such as the frontostriatal network in ADHD or the motor circuits in Tourette syndrome. Beyond local effects, TMS influences network-level connectivity, reshaping dysfunctional neural pathways [30]. Also, it has been shown to modulate neurotransmitter systems, such as increasing dopamine release in the striatum, which is critical for attentional and motor control [31].

## Applications in Neurodevelopmental Disorders

**Table:** Table1. Main Mechanisms of TES

**Mechanism**	**Description**	**Key Features**	**Modalities**
**Modulation of Membrane Potential**	Alters neuronal membrane potential, making neurons more or less likely to fire action potentials.	- Can induce excitability (depolarization) or inhibition (hyperpolarization). - Effects depend on polarity and current strength.	tDCS, tACS
**Neuroplasticity Induction**	Promotes long-term potentiation (LTP) or long-term depression (LTD) in synaptic connections.	- Activity-dependent changes. - Related to synaptic strengthening or weakening. - Can be sustained post-stimulation.	tDCS, tACS, tRNS
**Network Synchronization**	Modifies the synchronization of oscillatory activity between neural networks.	- Can enhance or disrupt cortical rhythms. - Frequency-specific effects.	tACS, tRNS
**Subthreshold Stimulation**	Influences neuronal activity without directly causing action potentials.	- Subtle effects on resting-state activity. - Alters spontaneous or evoked activity in the brain.	tDCS, tACS
**Stochastic Resonance**	Enhances signal transmission in noisy systems through the addition of random electrical input.	- Improves signal-to-noise ratio. - May enhance perception or motor performance.	tRNS
**Neurochemical Modulation**	Alters the release or uptake of neurotransmitters in the brain.	- Can affect dopamine, glutamate, and GABA activity. - Potential links to mood and cognitive improvements.	tDCS, tACS

tDCS: Transcranial Direct Current Stimulation. tACS: Transcranial Alternating Current Stimulation. tRNS: Transcranial Random Noise Stimulation

**Table:** Table2. Main Mechanisms of TMS

**Mechanism**	**Description**	**Key Features**	**Modalities**
**Induction of Electric Fields**	Creates an electric field in the brain via rapidly changing magnetic fields, causing depolarization of neurons.	Direct activation of neurons near the stimulation site. Localized effects. Strength depends on coil type and intensity.	Single-pulse TMS, rTMS
**Neuroplasticity Induction**	Modifies synaptic strength through LTP or LTD.	Changes in cortical excitability. Effects can last beyond stimulation. nfluences both local and connected areas.	rTMS, TBS
**Cortical Inhibition/Excitation**	Balances inhibitory and excitatory circuits depending on stimulation parameters.	Frequency-specific effects: Low (≤1 Hz): inhibitory. High (>5 Hz): excitatory.	rTMS, paired-pulse TMS
**Network Modulation**	Alters functional connectivity between brain regions by targeting specific nodes in neural networks.	Changes functional coupling. enhance or suppress communication in large-scale brain networks.	rTMS, TBS
**Plasticity via Hebbian Mechanisms**	Stimulates activity-dependent plasticity based on the principle of "neurons that fire together, wire together."	Repetition enhances synaptic changes. Timing-dependent effects (e.g., spike-timing-dependent plasticity).	PAS
**Subcortical Stimulation**	Indirectly affects deeper brain structures through cortical-subcortical connections.	Requires higher stimulation intensity.	rTMS

Single-pulse TMS: Single magnetic pulse to probe cortical excitability. rTMS: Repetitive TMS, delivers multiple pulses in trains to induce lasting effects. Theta Burst Stimulation (TBS): High-frequency bursts mimicking natural theta rhythms. Paired-pulse TMS: Two pulses with varied intervals to study cortical inhibition or facilitation. Paired Associative Stimulation (PAS): Combines TMS and peripheral nerve stimulation to induce plasticity.

**Table:** Table3. Comparison of TES and TMS Techniques

**Aspect**	**Transcranial Electrical Stimulation (TES) **	**Transcranial Magnetic Stimulation (TMS) **
**Target Depth**	Primarily superficial cortical regions near electrode placement.	Can target both superficial and deeper cortical structures.
**Effect on Plasticity**	Indirectly promotes LTP and LTD through modulation of synaptic activity and excitability.	Directly induces LTP or LTD by triggering action potentials and modifying synaptic strength.
**Temporal Precision**	Limited; continuous stimulation influences neural activity over time.	High; can achieve millisecond-level precision in neuronal activation.
**Oscillatory Modulation**	tACS can align endogenous oscillatory activity to stimulation frequency.	Modifies oscillatory activity depending on stimulation frequency and protocol (e.g., rTMS, TBS).
**Session Duration**	20-30 minutes per session; requires daily sessions over several weeks for significant effects.	15-40 minutes per session; fewer sessions needed due to more robust stimulation effects.
**Side Effects**	Mild (e.g., tingling, skin irritation, headache).	Mild to moderate (e.g., scalp discomfort, headache, rare risk of seizure).
**Cost and Accessibility**	Low-cost, portable, and suitable for home-based applications with supervision.	High cost; requires specialized equipment and trained professionals.
**Applications in NDDs**	Effective for mild to moderate symptoms and surface-level cortical modulation.	Effective for severe symptoms and deeper or more localized cortical targets.

**Table:** Table4. Application of TES/TMS in NDDs

**Disorder**	**Affected Brain Regions **	**TES Target**	**TMS Target**	**Key Findings**
ASD [13][14]	Prefrontal cortex, amygdala, temporal lobe	DLPFC, TPJ, Motor cortex (M1)	DLPFC, Left premotor cortex	**TES** improves phonological processing and reading fluency. **TMS**enhances reading comprehension and language processing.
ADHD [14]	Prefrontal cortex, basal ganglia	DLPFC	M1, DLPFC	**TES** improved attention, working memory, and reduced impulsivity. **TMS** improved inhibitory control, ADHD symptoms, and cognitive flexibility.
Tourette Syndrome [11][14]	Supplementary motor area, basal ganglia	Motor cortex	SMA	**TES**reduces tic severity. **TMS** reduced tic frequency and severity.
Dyslexia [14][53]	Left temporoparietal cortex	Left TPC,	LIFG, Left TPC	**TES** improves phonological processing and reading fluency. **TMS** enhances reading comprehension and language processing
DCD [56][70]	Motor cortex, cerebellum	Motor cortex (M1); SMA	M1 PFC	**TES** facilitates motor skill learning and coordination. **TMS** improves motor planning and execution, especially with high-frequency stimulation.
Intellectual Disabilities (ID) [60][75]	Global cortical and subcortical regions	DLPFC; Medial PFC	DLPFC; Right PFC	**TES** enhances cognitive flexibility and working memory. **TMS** improves problem-solving and attention, with potential neuroplasticity effects.

DLPFC: Dorsolateral Prefrontal Cortex; TPJ: Temporoparietal Junction; TPC:Temporoparietal Cortex; rIFG: Right Inferior Frontal Gyrus; PFC: Prefrontal Cortex; SMA: Supplementary Motor Area; lIFG: Left inferior frontal gyrus

The application of TES and TMS has shown promise in addressing core symptoms of neurodevelopmental disorders (NDDs) [6][10]. Table-3 makes comparison of TES and TMS techniques. Due to the lower cost of TES equipment, it is more accessible, leading to a greater volume of research on TES compared to TMS [32]. Table-4 present the application of TES/TMS in NDDs.

### 1.TES

#### 1. 1. Autism Spectrum Disorder (ASD)

ASD is characterized by deficits in social communication, repetitive behaviors, and restricted interests [33]. TES and TMS have been explored for their potential to improve social cognition, executive functioning, and behavioral regulation in ASD [13]. A notable application of TES in ASD involves targeting gamma-frequency oscillations, which are often disrupted in individuals with ASD due to inhibitory interneuron dysfunctions. Gamma oscillations are essential for neural communication and cognitive processes, and disruptions in these rhythms contribute to the social and cognitive deficits observed in ASD [34]. Kayarian et al. [35] highlighted the potential of TES, particularly tACS, to entrain gamma oscillations, thereby improving inhibitory signaling and mitigating gamma-related abnormalities observed in ASD. This approach could enhance cognitive and social functions by addressing underlying neural dysregulation. Another promising area of research is the application of anodal tDCS to improve specific behavioral and cognitive functions [36]. Nazari et al. [37] demonstrated that stimulating the left dorsolateral prefrontal cortex (DLPFC) with anodal tDCS improved facial emotion recognition (FER) in boys with ASD. Such advancements are critical, as FER deficits significantly impair social interactions in individuals with ASD.

Moreover, this study reported significant improvements in clinical symptom scales, further supporting the efficacy of tDCS as a complementary therapy. However, these findings are limited by small sample sizes and short follow-up durations, which reduce generalizability and the ability to assess long-term effects. [37]. Also, several studies reported over multiple sessions, the combination of tDCS and cognitive training leads to improvements in social functioning and cognitive processing speed in ASD [38][39]. Moreover, TES has been evaluated by Hupfeld et al [40] for its impact on motor and language planning in minimally verbal children with ASD. They showed that low-intensity anodal tDCS improved motor planning and grammar use in children, particularly when combined with speech and occupational therapies [40].

### 1 .2. Attention-Deficit/Hyperactivity Disorder (ADHD)

ADHD is a prevalent neurodevelopmental condition characterized by inattention, hyperactivity, and impulsivity which is often linked to Dysregulation in the frontostriatal circuitry that underpins core symptoms of ADHD, including impulsivity and difficulty with sustained attention [41].

Unlike tDCS, tRNS appears to provide more enduring benefits, likely due to its capacity to enhance synaptic plasticity across broader neural networks. tRNS has been increasingly investigated as a non-invasive and targeted approach for modulating neural activity associated with ADHD [12]. Recent research highlights the potential of TES to improve cognitive functions in individuals with ADHD. For example, a pilot study by Dakwar-Kawar et al.[21] demonstrated that tRNS combined with cognitive training significantly enhanced processing speed in children with ADHD, particularly under conditions of mental fatigue. Improvements were sustained for at least one week post-intervention, underscoring the potential long-term benefits of integrating TES with behavioral therapies [21].

TES has demonstrated efficacy in addressing cognitive deficits and core neural dysfunctions in ADHD. A systematic review by Salehinejad et al. [42] highlighted the effectiveness of tDCS in modulating the dorsolateral prefrontal cortex (dlPFC), a critical region for executive functioning and impulse control. Also, they emphasized the safety and tolerability of tDCS, making it a feasible adjunct to conventional therapies [42]. Furthermore, Boetzel et al. [43] discussed potential targets for TES in ADHD, focusing on the modulation of oscillatory patterns and connectivity in the prefrontal cortex. Their findings align with the hypothesis that ADHD involves dysregulated neural circuits that can be selectively influenced by electrical stimulation [43].

### 1. 3. Tourette Syndrome

Tourette Syndrome is a neurodevelopmental disorder characterized by involuntary motor and vocal tics, often associated with dysregulated activity in the Cortico-Striato-Thalamo-Cortical (CSTC) networks [44]. TES, particularly transcranial direct current stimulation (tDCS), has been explored as a non-invasive method to modulate aberrant neural activity and alleviate tics in Tourette Syndrome [11].

Several trials provide promising evidence for the therapeutic potential of TES in managing TS [11]. A clinical trial demonstrated that cathodal tDCS targeting the SMA might have the potential to reduce tic severity in TS. The study showed a significant decrease in tic impairment scores post-cathodal stimulation [45]. Also, a case report highlights the ability of cathodal tDCS to downregulate hyperactivity in CSTC circuits, potentially providing long-term relief from tics [19].

This application of TES aligns with the understanding that tics arise from hyperexcitable cortical regions and dysfunctional inhibitory control mechanisms [46]. Cathodal tDCS, by decreasing cortical excitability, offers a targeted approach to restore balance in these neural networks. Preliminary evidence suggests that the efficacy of cathodal tDCS may vary depending on tic severity, with more pronounced benefits observed in individuals with mild to moderate symptoms. [19]. However, the mechanisms underlying its effects remain to be fully elucidated, emphasizing the need for randomized controlled trials to optimize stimulation protocols and verify its efficacy across diverse TS populations [11].

In addition to its direct therapeutic effects, TES serves as a valuable tool for investigating the neurophysiology of TS. It offers insights into the dynamics of CSTC networks and how modulation of specific brain regions correlates with symptom alleviation [47].

### 1. 4. Other Neurodevelopmental Disorders

TES’s application in conditions like dyslexia, developmental coordination disorder (DCD), and intellectual disabilities highlights its versatility in modulating neural pathways to address specific cognitive and motor deficits. These interventions may have limited generalizability, particularly in older populations or those with co-occurring learning disabilities [48][49][50].

Dyslexia, a neurodevelopmental disorder affecting reading and phonological processing, has been consistently associated with atypical neural activity in the temporoparietal and frontal brain regions [51][52].

Marchesotti et al. [53] The focal intervention targeting the left auditory cortex was found to reduce 30-Hz activity in the right superior temporal cortex, thereby restoring left-hemisphere dominance in oscillatory responses. This outcome provides evidence for the causal involvement of neural oscillations in phonological processing. Furthermore, these findings present a robust neurophysiological basis for addressing low-gamma anomalies and potentially mitigating the phonological deficits associated with dyslexia [53]. Also, another experimental research demonstrated that tACS applied at 40 Hz to the auditory cortex significantly enhanced phoneme-categorization abilities in individuals with developmental dyslexia [48]. They revealed that the improvements in auditory temporal resolution were associated with increased amplitudes of the P50-N1 complex, a key marker of sensory processing efficiency in the auditory system. These findings underscore the potential of tACS as a novel intervention for auditory and linguistic impairments in dyslexia. [48].

Developmental coordination disorder (DCD), a condition associated with impairments in motor learning, arises from dysfunctions within motor and cerebellar neural networks. These disruptions affect the coordination and execution of motor skills, leading to challenges in fine and gross motor performance [54]. TES is being evaluated for its potential to enhance motor planning and execution by targeting the primary motor cortex and supplementary motor area (SMA) [55]. Cathodal stimulation has been particularly effective in reducing overactivity in motor regions, thereby improving coordination and reducing error rates in motor tasks [56]. Combined interventions that integrate TES with physical or occupational therapy are being examined to optimize motor outcomes [57].

Intellectual disabilities, A disorder characterized by broad cognitive deficits, encompassing challenges in executive functioning, attentional regulation, and information processing speed [58]. TES provides a promising approach to addressing global cognitive delays and executive dysfunctions [59].

Neurophysiological studies indicate that deficits in prefrontal cortex activity contribute significantly to the cognitive and behavioral challenges observed in individuals with intellectual disabilities [60].

Overall, tDCS presents a promising strategy for improving processing speed in children with intellectual disabilities. Through its ability to modulate neural activity and mitigate symptoms of mental health and neurological challenges, tDCS offers a pathway to enhancing cognition and fostering academic and social development [50][59].

### 2.TMS

#### 2.1.ASD

Recent studies have explored the potential of repetitive TMS (rTMS) in improving social and communication skills in individuals with ASD [61]. For example, Kaokhieo et al. [62] showed the feasibility of combining rTMS with action-observation-execution training to enhance social interaction and communication. This approach leverages rTMS-induced plasticity in motor and mirror neuron systems, which are believed to be implicated in social cognitive deficits in ASD [62]. Furthermore, Yang et al. [63] reported that rTMS modulate long-range functional connectivity, potentially restoring balance between hyper- and hypoconnectivity in brain networks linked to ASD symptoms. These findings align with theoretical frameworks suggesting that ASD involves atypical neural network organization [63]. Moreover, TMS metrics, such as cortical excitability and inhibition, can serve as rapid, non-invasive biomarkers for ASD. Such markers could aid in tailoring interventions to individual neurophysiological profiles, optimizing therapeutic outcomes [64].

### 2.2.ADHD

Santos et al.[28] proposed a framework in which rTMS targets prefrontal regions to enhance dopamine signaling and adjust disrupted circadian patterns. They suggest that rTMS could offer a multifaceted approach to ADHD treatment by addressing both behavioral symptoms and underlying neurophysiological mechanisms [28].

A notable development involves the exploration of TMS as a diagnostic and therapeutic tool for assessing cortical inhibition, which is often reduced in ADHD [65]. TMS-evoked EEG responses, such as the N100 component, have been identified as potential biomarkers of cortical dysfunction [66][67].

### 2.3.Tourette Syndrome

The repetitive TMS (rTMS) in modulating the supplementary motor area (SMA), a critical region implicated in tic generation and control [27], although, a recent meta-analysis revealed that while TMS does not significantly decrease tic severity, it has a moderate and statistically significant effect on reducing premonitory urge severity in Tourette syndrome [68]. Kahl et al. [69] showed bilateral rTMS of the SMA in children with Tourette syndrome effectively reduced tic severity. Their open-label clinical trial highlighted not only the feasibility and safety of the procedure but also its physiological impact, suggesting enhanced inhibitory control within motor networks as a potential mechanism underlying symptom improvement.

### 3.Other Neurodevelopmental Disorders

• Dyslexia: Studies utilizing high-frequency TMS over the left temporoparietal cortex have reported improved phonological decoding and word recognition skills [70]. Initial trials have demonstrated that repeated sessions of TMS can normalize activity in dyslexia-affected regions, enhancing reading-related neural circuitry [71].

• DCD: To enhance motor learning and coordination, TMS interventions in DCD have primarily focused on motor areas, such as the primary motor cortex (M1) and the cerebellum [72]. rTMS has demonstrated effectiveness in enhancing motor activity and executive functions in neurodevelopmental contexts [73] and improving symptom-specific outcomes in conditions like spastic cerebral palsy [74].

• Intellectual Disabilities: TMS works by using electromagnetic pulses to stimulate or inhibit neuronal activity in targeted brain areas, thereby facilitating neural plasticity [75]. Although specific research on TMS for Intellectual Disabilities is limited, broader studies in neurodevelopmental and cognitive disorders suggest its efficacy in improving motor and cognitive functions. For example, TMS has been shown to enhance cortical excitability and facilitate learning processes in related contexts, supporting its potential as an adjunctive therapy [76][77].

## Challenges and Limitations

Despite the promising potential of TES and TMS for treating neurodevelopmental disorders (NDDs), several challenges and limitations hinder their widespread adoption and consistent efficacy[11]. One major limitation is the heterogeneity of NDDs, both in terms of symptom presentation and underlying neural dysfunction. For instance, children with ADHD who exhibit predominantly inattentive symptoms may respond differently to TES compared to those with hyperactive-impulsive presentations, necessitating tailored protocols [10][37].

Disorders like autism spectrum disorder (ASD) and attention-deficit/hyperactivity disorder (ADHD) encompass diverse phenotypes, making it difficult to design one-size-fits-all protocols [14]. The variability in stimulation outcomes is further compounded by individual differences, such as age, sex, genetic factors, and baseline cortical excitability, which influence the brain's responsiveness to stimulation (Sabé et al., 2024). This variability highlights the need for personalized approaches, yet developing tailored protocols remains challenging due to a lack of reliable biomarkers to predict treatment response [45].

Methodological issues also pose significant challenges. Studies in this field often suffer from small sample sizes, insufficient control groups, and heterogeneity in stimulation parameters, including intensity, duration, frequency, and target areas [68].

The lack of standardized stimulation protocols complicates cross-study comparisons and hinders reproducibility, presenting a significant barrier to advancing the field [10]. Additionally, while TES is relatively easy to implement, its effects are often superficial and limited to cortical regions near the electrodes. This limitation may reduce its efficacy for disorders involving deeper brain structures [48]. On the other hand, while TMS can target deeper regions and induce more robust changes in neural activity, it requires expensive equipment, trained personnel, and specialized facilities, restricting its accessibility and scalability, so developing portable, low-cost TES devices and training community health workers could improve accessibility in low-resource settings[14][32].

Ethical concerns are particularly pronounced in pediatric populations, the primary demographic affected by NDDs. The long-term effects of repeated stimulation on the developing brain are not fully understood, raising questions about the safety and appropriateness of these interventions in children and adolescents [78][79]. While TES is generally well-tolerated, TMS carries risks such as headaches, discomfort, and in rare cases, seizures, which necessitate careful screening and monitoring (Kahl et al., 2021).

Furthermore, the psychological impact of these interventions on young patients, including potential stigma or stress related to undergoing brain stimulation, must be carefully considered [10]. To address these concerns, rigorous safety monitoring, parental consent, and age-appropriate protocols are essential for ensuring ethical application [79].

Lastly, there is a significant need for longitudinal studies to determine the durability of the therapeutic effects of TES and TMS in NDDs. Most existing studies focus on short-term outcomes, leaving gaps in understanding whether these interventions lead to lasting improvements or require ongoing maintenance [68]. Without long-term data, it remains unclear how these therapies influence developmental trajectories or whether repeated use could lead to diminishing returns or unintended consequences [45]. Addressing these challenges through rigorous research, improved standardization, and ethical oversight will be crucial to unlocking the full potential of TES and TMS in the treatment of neurodevelopmental disorders. These studies should prioritize outcomes such as sustained cognitive improvements, enhanced quality of life, and reduced symptom severity over time [14].

## Future Perspectives

The application of TES/TMS in NDDs presents significant potential, yet several areas remain unexplored, warranting future investigation [13]. Emerging biomarkers, such as alpha-band EEG activity, could guide the personalization of stimulation parameters to maximize therapeutic efficacy[67][80]. Personalized approaches that account for developmental differences, symptom severity, and cortical anatomy are likely to improve therapeutic outcomes [67][73].

Integrating TES and TMS with complementary therapies, such as behavioral interventions, pharmacological treatments, or educational programs, holds the potential for synergistic effects, enhancing therapeutic outcomes [11][13]. For example, pairing stimulation with cognitive-behavioral therapy may facilitate neural plasticity, leading to greater improvements in executive functioning and emotional regulation [38][39]. Additionally, the exploration of emerging techniques, such as closed-loop stimulation systems that adapt stimulation parameters in real time based on neural activity, could offer a more dynamic and responsive approach to treatment [81].

While current studies have demonstrated the short-term benefits of NIBS, understanding its long-term effects remains a critical challenge. Longitudinal studies are essential to assess the durability of therapeutic gains and identify any potential risks associated with repeated or prolonged use, particularly in pediatric populations where brain development is ongoing [11]. These studies should also explore how early interventions with TES or TMS might alter developmental trajectories and potentially prevent the worsening of symptoms over time [10][11].

Expanding research into new disorders and underrepresented groups is crucial. While much of the current work focuses on autism, ADHD, and Tourette's, other conditions like dyslexia, developmental coordination disorder, and intellectual disabilities remain understudied [5]. Broadening the scope of research will help determine if NIBS can benefit these less-studied conditions. Ensuring diversity in study populations is also important, as cultural, genetic, and environmental factors may impact the effectiveness and tolerability of NIBS interventions [11][13].

Finally, the ethical considerations of using NIBS in pediatric populations must remain at the forefront of future research. These studies should prioritize outcomes such as sustained cognitive improvements, enhanced quality of life, and reduced symptom severity over time. [14].Establishing robust ethical guidelines and conducting comprehensive safety evaluations will be critical to ensuring that the benefits of these technologies outweigh the risks[11].

## Conclusion

The use of TES and TMS represents a promising frontier in the treatment of NDDs, offering non-invasive methods to modulate neural circuits and improve core symptoms such as attention deficits, social communication challenges, and motor dysfunctions. Despite significant advancements, the current evidence base is characterized by variability in study designs, inconsistent outcomes, and limited understanding of the long-term effects of these interventions. However, the existing literature provides compelling evidence for their potential as adjunctive therapies, particularly in conditions such as ASD, ADHD, and Tourette syndrome.

TES's ease of use, safety profile, and cost-effectiveness make it particularly suitable for managing mild to moderate symptoms, with the added potential for supervised home-based applications. This accessibility, compared to TMS, makes TES more appealing and intriguing to researchers. On the other hand, TMS with its ability to directly target deeper cortical and subcortical structures, appears more effective for severe and persistent symptoms. Despite these strengths, challenges related to scalability, particularly for TMS, require innovative solutions to broaden its accessibility. Both techniques benefit from their ability to enhance neuroplasticity and modulate network activity, though further research is needed to optimize protocols.

Future directions should prioritize the integration of NIBS with other therapeutic modalities, such as behavioral and pharmacological interventions, to achieve synergistic effects. Longitudinal studies are critical to elucidate the durability of effects, safety in pediatric populations, and the potential developmental impacts of repeated stimulation. Multi-center collaborations involving diverse populations will be critical for generating robust, generalizable data on the long-term effects of these interventions. Moreover, addressing ethical considerations, improving standardization of protocols, and expanding research to include underrepresented NDD populations will be essential to advance the field.

Overall, TES and TMS offer innovative avenues for improving outcomes in NDDs, transforming how these complex conditions are managed. Continued multidisciplinary efforts in research and clinical practice will be key to unlocking the full potential of these technologies and ensuring they become accessible, effective, and safe tools for individuals across the lifespan.

## Conflict of Interest

None.
